# Nitrous Oxide: No More a Laughing Matter

**DOI:** 10.7759/cureus.85467

**Published:** 2025-06-06

**Authors:** Amit Badshah, Abir Aijaz, Abdul Bhat, Ayesha Babar

**Affiliations:** 1 Acute and General Internal Medicine, Weston General Hospital, University Hospitals Bristol and Weston, Weston-super-Mare, GBR; 2 Acute Medicine, Weston General Hospital, University Hospitals Bristol and Weston, Weston-super-Mare, GBR

**Keywords:** ataxia, canister, folate, hydroxocobalamin, inhalation, nitrous oxide, spinal cord, spinal mri, subacute combined degeneration of the spinal cord, vitamin b12

## Abstract

Nitrous oxide (N₂O) is an uncommon cause of subacute combined degeneration (SCD) of the spinal cord, typically observed in district general hospitals, with most affected patients being male. A genetic predisposition may contribute to the development of this myelopathy, particularly due to polymorphisms in the gene responsible for vitamin B12 processing. Our case is distinct in its presentation, involving prolonged exposure over two years, thoracic dorsal column involvement, and rapid, significant recovery within two months.

## Introduction

Subacute combined degeneration (SCD) of the spinal cord is a well-recognized but uncommon neurological disorder caused primarily by vitamin B12 deficiency. It leads to progressive demyelination of the dorsal (posterior) and lateral columns of the spinal cord. The affected tracts are responsible for proprioception, vibration sense, and motor function. While pernicious anemia and malabsorption remain the most common causes, nitrous oxide(N₂O) inhalation abuse is an increasingly reported, though still uncommon, etiology, with a noted male predominance [1]. N₂O, commonly referred to as “laughing gas,” has legitimate applications in anesthesia and dentistry, but its recreational use is on the rise, particularly among young adults. Although often perceived as harmless, chronic use can lead to serious neurological consequences, most notably SCD of the spinal cord, a potentially irreversible condition caused by functional or absolute vitamin B12 deficiency [2].

This case highlights the clinical presentation, features, and pathophysiology of N₂O-induced SCD in a young patient with acute-on-chronic recreational use. N₂O inactivates vitamin B12​​​​​​​ by oxidizing its cobalt core, thereby impairing methionine synthase activity and ultimately disrupting myelin synthesis. Despite normal serum B12​​​​​​​ levels on routine testing, patients may still exhibit a functional deficiency, underscoring the importance of measuring methylmalonic acid (MMA) and homocysteine levels in suspected cases. SCD primarily affects the dorsal columns and lateral corticospinal tracts. The absence of bladder or bowel involvement and preserved cranial nerve function helped localize the pathology to the posterior and lateral spinal cord [3].

While the recreational use of N₂O is often overlooked, its popularity is rising due to its easy availability and brief euphoric effects. Chronic users are at high risk for neurological complications, especially when large quantities are consumed over a short period. Emerging evidence suggests a genetic predisposition due to polymorphisms in vitamin B12​​​​​​​ metabolism genes, which may increase susceptibility to N₂O-induced myelopathy [4].

## Case presentation

We present the case of a 23-year-old woman who presented to the emergency department with a one-week history of worsening numbness, paresthesia, and hyperesthesia in her arms, legs, and torso. The neurological symptoms initially presented in the lower limbs and progressed in an ascending manner. She did not report any bladder or bowel complaints, and both systems remained intact throughout the course of her treatment. She noticed extreme difficulty in walking and had an unsteady gait. She also described her walking as if walking on a soft cotton wool-like surface. Past medical history included mixed anxiety and depression, a previous suicide attempt, and an intentional drug overdose. The patient did not report any visual disturbances, headache, seizure activity, or disturbances in higher mental functions. There was no history of any preceding viral or bacterial infection, vaccination, head or spinal trauma, or gastroenteritis. She was not known to have neuropathy, diabetes mellitus, or any other metabolic illness that could explain the sudden onset of the neurological symptoms. She was a non-smoker, and her alcohol intake was moderate. The patient was otherwise fully independent, with a WHO performance status of zero, and worked in a shipping company. Her medications included sertraline and a combined oral contraceptive pill.

On further inquiry, the patient admitted to inhaling from 30 cans of N₂O at a party one week ago. Further history also revealed that she had been inhaling N_2_O regularly for two years. The frequency of inhalation ranged from once to twice per month, with 10-15 cans on each occasion. On examination, she had a Glasgow Coma Scale (GCS) score of 15. Blood pressure was 120/82 mmHg, temperature 36.7°C, and heart rate 92 beats per minute. Cardiovascular, respiratory, and abdominal examinations were entirely normal.

**Figure 1:**
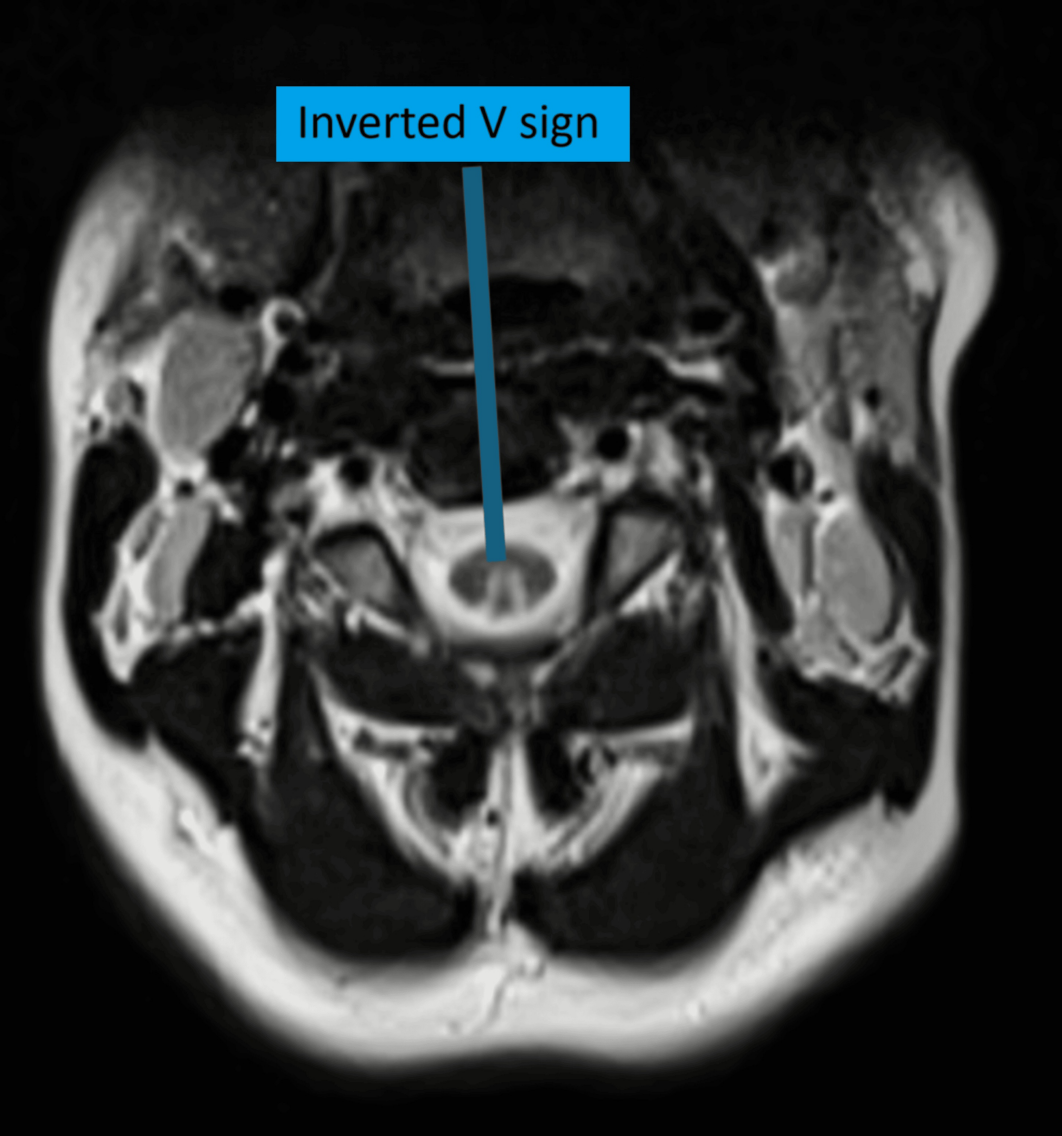
Inverted “V” or “rabbit ear” sign on axial T2-weighted MRI

**Figure 2:**
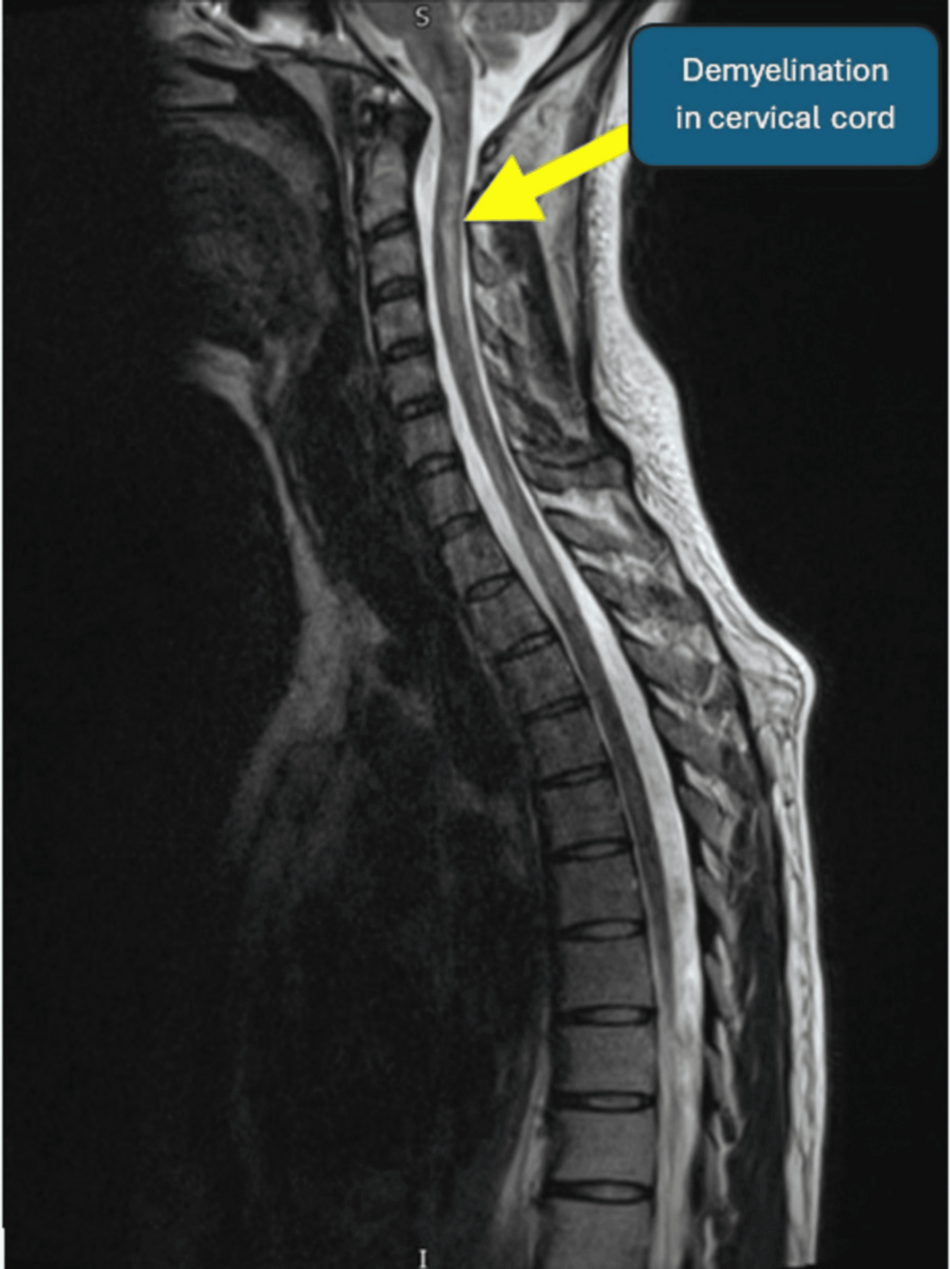
MRI of the spine showing demyelination in the cervical spine

A detailed neurological examination in the emergency department revealed normal muscle power (5/5) in both upper limbs, while the power in both lower limbs was reduced to 4/5. Cranial nerve examination was normal. Sensations of crude touch, fine touch, and pain were preserved. However, the patient reported paresthesias, describing the sensation of skin contact as “Someone had touched a funny bone.” Both ankle reflexes were absent, and plantar responses were withdrawal. What was initially thought to be horizontal nystagmus was later identified as gaze-evoked nystagmus. The patient's gait was unsteady, and the Romberg sign was positive. She was barely able to walk a few steps without assistance. Joint position sense and proprioception were severely impaired up to the knees. Knee reflexes were diminished, though upper limb reflexes remained intact. Further investigations included whole spine MRI and blood tests (Figures 1, 2; Tables 1, 2).

**Table 1:** CSF results of the patient

Parameter	Result	Reference range	Unit
White cells	<1	0-5	cells/μL
Glucose	3.4	2.4-4.4	mmol/L
Protein	0.28	0.15-0.45	g/L
Virology	Negative		

**Table 2:** Blood tests on admission HB: hemoglobin, MCV: mean corpuscular volume, TSH: thyroid-stimulating hormone.

Test	On admission	Third day	Reference range
HB	128	118	120-150 g/L
MCV	100.3	100.5	80-100 f/L
Platelet	460	401	109/L
B_12_	341	>2000	180-900 ng/L
Folate	14.5	20	2.5-19.5 µg/L
Homocysteine	144.2	8.8	2-11.3 μmol/L
Methylmalonic acid	284		0-280 nmol/L
TSH	1.37		0.27-4.2 mIU/L
Creatinine	67		45-84 μmol/L
Ceruloplasmin	0.27		0.23-0.60 g/L
HbA1c	33		<42 mmol/mol
Copper	16		11-25 mmol/L

Treatment

As there were no local trust or regional guidelines for managing N₂O-induced SCD of the spinal cord, the patient was treated based on advice from the National Poisons Information Service. She received hydroxocobalamin 1 mg intramuscularly daily for one week, followed by weekly injections for four weeks, and then monthly for one year. She was also enrolled in a regular physiotherapy program, which significantly contributed to her overall recovery. At the one-month follow-up, the patient reported substantial improvement in her symptoms and was able to walk independently with minimal assistance using a walking stick.

## Discussion

N₂O, also known as laughing gas, is a sweet-smelling, colorless gas first discovered in 1772. It has been used as a dissociative anesthetic for over a century. Apart from its medical applications, recreational use of N₂O has become increasingly common, largely due to its legal over-the-counter availability [5]. Currently, N₂O abuse ranks as the second most commonly used recreational drug among young people in the UK [6].

The neurological complications associated with N₂O, such as SCD of the spinal cord, were first documented in 1978, when 14 dentists were identified as having N₂O-induced SCD [7].

Whipped cream chargers, or “whippets,” commonly sold in head shops, are typically fitted onto dispensers that release N₂O into balloons for recreational inhalation. The psychotropic effects peak within approximately 20 seconds and typically subside around two minutes. The hazardous consequences of N₂O abuse, most notably SCD of the spinal cord, are due to inactivation of vitamin B12. N₂O oxidizes the cobalt ion at the core of vitamin B12, rendering it inactive. This impairs its role as a cofactor in essential biochemical processes, particularly the recycling of homocysteine to methionine via methionine synthase, ultimately leading to demyelination [8].

Vitamin B12 deficiency leads to SCD of the spinal cord by disrupting biochemical processes essential for myelin maintenance. B12 is crucial for the conversion of homocysteine to methionine (via methylcobalamin), a reaction necessary for the synthesis of S-adenosylmethionine (SAM), a major methyl donor in myelin formation. It also facilitates the conversion of methylmalonyl-CoA to succinyl-CoA (via adenosylcobalamin); without B12​​​​​​​, MMA accumulates, exerting toxic effects on myelin-producing cells. These combined disruptions result in demyelination, particularly affecting the posterior and lateral columns of the spinal cord [8,9].

Normally, activated vitamin B12​​​​​​​ is essential for myelin synthesis and overall neuronal health. The two active forms, methylcobalamin and adenosylcobalamin, play distinct but complementary roles. Methylcobalamin serves as a cofactor in the remethylation of homocysteine to methionine, which is subsequently converted to SAM, a major methyl group donor crucial for the synthesis of phospholipids in the myelin sheath. Adenosylcobalamin is a cofactor for methylmalonyl-CoA mutase, the enzyme responsible for converting methylmalonyl-CoA to succinyl-CoA. In vitamin B12​​​​​​​ deficiency, excess methylmalonyl-CoA accumulates and is incorporated into fatty acid synthesis instead of malonyl-CoA. This results in structurally unstable myelin, rendering it prone to demyelination [9]. The diagnosis of N₂O-induced SCD requires a detailed clinical history, high index of suspicion, thorough neurological examination, and correlation with laboratory markers. Spinal MRI is the imaging of choice to confirm dorsal column involvement. Importantly, normal serum B12​​​​​​​ levels do not exclude functional deficiency, as inactivation by N₂O can impair its biochemical activity despite adequate circulating levels [10].

Epidemiology

Most patients presenting with N₂O-induced SCD are between the ages of 15 and 30 years, with males comprising approximately 75% of reported cases. Interestingly, although females represent only 25% of cases, they appear to be at an increased risk of developing SCD at higher doses of N₂O exposure. Ethnic background is also postulated to have a role in susceptibility, with a higher incidence noted among individuals of Bangladeshi descent and certain Dutch populations [10,11].

Identified risk factors for N₂O-induced SCD include (1) high-volume use (e.g., >20 canisters per session), (2) prolonged use (i.e., >6 weeks), and (3) pre-existing subclinical vitamin B12 deficiency.

Clinical features

The classical signs and symptoms of SCD may manifest from a few days up to six months following N₂O exposure. Notably, the lag period after even a single exposure to N₂O can be as short as 2-6 weeks [12,13]. The most common presenting symptom is distal paresthesia of the lower limbs, with or without upper limb involvement, which is often dismissed by patients. However, it can progress to more serious symptoms such as ataxia, gait disturbance, and frequent falls [14]. A minority of patients may present with muscle weakness, urinary or fecal incontinence, Lhermitte's sign (a sudden electric shock-like sensation radiating down the spine), sexual problems/dysfunction, myoclonus, or even psychiatric symptoms [15]. A rare but serious presentation includes sudden-onset shortness of breath (SOB) with desaturation, possibly due to pulmonary embolism, or leg swelling from deep vein thrombosis (DVT). These may result from N₂O-induced hyperhomocysteinemia. Hyperhomocysteinemia is a well-known risk factor for thrombosis [16], and homocysteine levels are often evaluated when investigating thrombotic events in young individuals.

The key challenge in managing N₂O-associated SCD lies in the early identification and awareness of risk factors among clinicians, enabling timely suspicion and diagnosis. These risk factors include dietary habits, history of gastrointestinal surgeries, chronic gastrointestinal disorders such as inflammatory bowel disease (IBD) or malabsorption syndromes like celiac disease, and medications known to impair vitamin B12 absorption, including proton pump inhibitors (PPIs), metformin, oral contraceptive pills (OCPs), and potassium supplements [17]. On clinical examination, findings are often symmetrical and typically involve both corticospinal and dorsal column dysfunction. Common signs include loss of vibration and proprioception, positive Romberg’s sign, positive Babinski reflex, and absent ankle jerk [18,19].

Approach to diagnosis and workup

Whenever a suspected case of N₂O-induced SACD is encountered, it warrants a thorough and detailed history, with particular attention to the duration and quantity of N₂O use. This should be followed by a meticulous neurological examination, focusing on posterior column signs such as impaired proprioception and vibration sense.

Neurology consultation is advised if the clinical presentation raises suspicion of an alternative diagnosis. These include vision disturbances, recent antecedent illness, ascending symptoms (as opposed to the typical descending pattern), dysautonomia, back pain, fever, or a history suggestive of malignancy.

Laboratory investigations should include full blood count (FBC), liver function tests (LFTs), thyroid function tests (TFTs), urea and electrolytes (U&E), folate, MMA, and homocysteine levels to support a presumptive diagnosis and confirm vitamin B12 deficiency.

Spinal imaging, preferably MRI of the cervical and thoracic spine, is recommended to document the extent of spinal cord involvement.

Following this, treatment should be initiated promptly to prevent irreversible neurological damage. The patient should receive high-dose hydroxocobalamin, although treatment protocols may vary by institution. In our case, the regimen followed was based on guidance from the UK National Poisons Information Service.

Lab investigations in N₂O-induced SCD

Serum vitamin B12 is the initial and most commonly performed test in suspected cases; however, levels may appear normal in N₂O-induced SACD, which represents a functional rather than absolute B12 deficiency, unlike in pernicious anemia. The sensitivity of serum B12 testing is limited, ranging between 20% and 50%. In contrast, MMA levels typically rise in N_2_O-associated SACD due to impaired vitamin B12​​​​​​​ activity, with a sensitivity of 80%-90%. Measurement of homocysteine, alongside folate levels, is also essential and serves as a specific marker for vitamin B12 deficiency, with sensitivity approaching 90%. Mean corpuscular volume (MCV) and hemoglobin (HB) are often normal but may be elevated in some cases. HIV and syphilis serology is important to exclude alternative diagnoses with similar presentations, such as tabes dorsalis. Copper and zinc levels should be assessed to rule out hypocupremia myelopathy. In addition, anti-intrinsic factor antibodies are required to rule out pernicious anemia. Measurement of vitamin D levels is advised to help exclude other metabolic or nutritional causes of myelopathy [20].

Imaging

MRI of the cervical and thoracic spinal cord plays a very crucial role in establishing the diagnosis of SCD and in excluding other potential causes of myelopathy. The most characteristic MRI finding is T2-weighted hyperintensity in the dorsal columns, most commonly between C3 and C5, seen in approximately 50%-100% of cases. The "rabbit ear," visible on axial T2-weighted images, is considered a classical radiological hallmark of SCD. Thoracic cord involvement is less frequently observed but can occur. Routine brain MRI or FLAIR is not routinely recommended.

Following detailed history, neurological examination, and imaging, the differential diagnosis includes Guillain-Barré syndrome, HIV-associated myelopathy, hypocupremia myelopathy, methotrexate-induced neurotoxicity, tabes dorsalis, pernicious anemia, and N₂O-induced SCD of the spinal cord.

## Conclusions

N₂O is an uncommon cause of SCD of the spinal cord, with the majority of reported cases occurring in male patients. A genetic predisposition has also been suggested, potentially due to polymorphisms in genes involved in vitamin B12 processing. This case is distinct due to several key factors: prolonged N₂O exposure over two years, thoracic dorsal column involvement, and rapid and significant clinical recovery within two months following prompt initiation of high-dose vitamin B12 therapy. The patient was closely monitored over subsequent months, including a follow-up MRI and lumbar puncture during admission. These investigations helped exclude other differential diagnoses, such as Guillain-Barré syndrome and neurosyphilis.
